# Interleukin-18 and IL-18 Binding Protein

**DOI:** 10.3389/fimmu.2013.00289

**Published:** 2013-10-08

**Authors:** Charles A. Dinarello, Daniela Novick, Soohyun Kim, Gilles Kaplanski

**Affiliations:** ^1^Department of Medicine, University of Colorado Denver, Aurora, CO, USA; ^2^Department of Medicine, University Medical Center Nijmegen, Nijmegen, Netherlands; ^3^Department of Molecular Genetics, Weizmann Institute of Science, Rehovot, Israel; ^4^Department of Biomedical Science and Technology, Konkuk University, Seoul, Republic of Korea; ^5^UMR-S 1076, Aix Marseille Université, Marseille, France; ^6^Service de Médecine Interne, Hôpital de la Conception, Assistance Publique Hôpitaux de Marseille, Marseille, France

**Keywords:** inflammation, autoimmune diseases, inflammasomes, interleukin-1, macrophages

## Abstract

Interleukin-18 (IL-18) is a member of the IL-1 family of cytokines. Similar to IL-1β, IL-18 is synthesized as an inactive precursor requiring processing by caspase-1 into an active cytokine but unlike IL-1β, the IL-18 precursor is constitutively present in nearly all cells in healthy humans and animals. The activity of IL-18 is balanced by the presence of a high affinity, naturally occurring IL-18 binding protein (IL-18BP). In humans, increased disease severity can be associated with an imbalance of IL-18 to IL-18BP such that the levels of free IL-18 are elevated in the circulation. Increasing number of studies have expanded the role of IL-18 in mediating inflammation in animal models of disease using the IL-18BP, IL-18-deficient mice, neutralization of IL-18, or deficiency in the IL-18 receptor alpha chain. A role for IL-18 has been implicated in several autoimmune diseases, myocardial function, emphysema, metabolic syndromes, psoriasis, inflammatory bowel disease, hemophagocytic syndromes, macrophage activation syndrome, sepsis, and acute kidney injury, although in some models of disease, IL-18 is protective. IL-18 plays a major role in the production of interferon-γ from T-cells and natural killer cells. The IL-18BP has been used safely in humans and clinical trials of IL-18BP as well as neutralizing anti-IL-18 antibodies are in clinical trials. This review updates the biology of IL-18 as well as its role in human disease.

## Introduction to IL-18

Interleukin-18 (IL-18) was first described in 1989 as “IFNγ-inducing factor” isolated in the serum of mice following an intraperitoneal injection of endotoxin. Days before, the mice had been pretreated with *Propionibacterium acnes*, which stimulates the reticuloendothelial system, particularly the Kupffer cells of the liver. Many investigators concluded that the serum factor was IL-12. With purification from mouse livers and molecular cloning of “IFNγ-inducing factor” in 1995 (1), the name was changed to IL-18. Surprisingly, the new cytokine was related to IL-1 and particularly to IL-1β. Similar to IL-1β, IL-18 is first synthesized as an inactive precursor and without a signal peptide, remains as an intracellular cytokine. The tertiary structure of the IL-18 precursor is closely related to the IL-37 precursor and the intron-exon borders of the IL-18 and IL-37 genes suggest a close association. Since 1995, many studies have used neutralization of endogenous IL-18 or IL-18-deficient mice to demonstrate the role for this cytokine in promoting inflammation and immune responses [reviewed in Ref. (2–4)]. However, the biology of IL-18 is hardly the recapitulation of IL-1β. There are several unique and specific differences between IL-18 and IL-1β. For example, in healthy human subjects and also in healthy mice, gene expression for IL-1β in blood mononuclear cells and hematopoietic cells is absent and there is no evidence that the IL-1β precursor is constitutively present in epithelial cells (5). In contrast, the IL-18 precursor is present in blood monocytes from healthy subjects and in the epithelial cells of the entire gastrointestinal tract. Peritoneal macrophages and mouse spleen also contain the IL-18 precursor in the absence of disease (5). The IL-18 precursor is also present in keratinocytes and nearly all epithelial cells. In this regard, IL-18 is similar to IL-1α and IL-33.

## Production and Activity of IL-18

### Processing of the IL-18 precursor by caspase-1

The IL-18 precursor has a molecular weight of 24,000 and is processed by the intracellular cysteine protease caspase-1, which cleaves the precursor into an active mature molecule of 17,200. As with the processing of IL-1β, inactive pro-caspase-1 is first activated into active caspase-1 by the nucleotide-binding domain and leucine-rich repeat pyrin containing protein-3 (NLRP3) inflammasome. Following cleavage by active caspase-1, mature IL-18 is secreted from the monocyte/macrophage, although over 80% of the IL-18 precursor remains unprocessed inside the cell. Compared to wild-type mice, mice deficient in caspase-1 do not release circulating IFNγ following endotoxin. IL-12-induced IFNγ is also absent in caspase-1-deficient mice (6). Importantly, any phenotypic characteristic of caspase-1-deficient mice must be studied as whether the deficiency is due to reduced IL-1β or IL-18 activity. For example, the caspase-1-deficient mouse is resistant to colitis (7) but the IL-1β-deficient mouse is susceptible in the same disease model (8). Since neutralizing antibodies to IL-18 are protective in the dextran sodium sulfate (DSS) colitis model, caspase-1 deficiency appears to prevent processing of IL-18 (7, 9). On the other hand, there are examples where caspase-1 processing of IL-18 is not required. For example, Fas ligand (FasL) stimulation results in release of biologically active IL-18 in caspase-1-deficient murine macrophages (10).

Similar to IL-1α and IL-33, the IL-18 precursor is constitutively expressed in endothelial cells, keratinocytes, and intestinal epithelial cells throughout the gastrointestinal tract. Macrophages and dendritic cells are the primary sources for the release of active IL-18, whereas the inactive precursor remains in the intracellular compartment of mesenchymal cells. Also, similar to IL-1α and IL-33, the IL-18 precursor is released from dying cells and processed extracellularly, most likely by neutrophil proteases such as proteinase-3 (11).

Although Fas signaling triggers apoptosis, Fas signaling induces inflammatory cytokine production, including IL-18. In addition to inducing IL-18, Fas signaling activates caspase-8 in macrophages and dendritic cells, which results in processing and release of mature IL-1β and IL-18 (12). It was also reported that the processing of IL-1β and IL-18 takes place independently of NLRP3 or RIP3 (12).

### Processing and secretion of the IL-18 precursor by ADAM 33-mediated VEGF-dependent mechanism

Because IL-18 stimulates vascular endothelial cells and promotes metastatic tumor cell invasion, studies had examined the mechanisms of IL-18 secretion from gastric cancer cell line. Vascular endothelial cell growth factor-D (VEGF-D) increased the expression as well as the secretion of IL-18 from the gastric cancer cell line (13). Since VEGF-D has a metalloprotease domain, knock-down of ADAM33 was examined and prevented the secretion of IL-18. Moreover, cell proliferation was reduced using ADAM33 small interfering RNA transfectants (13).

### Signal transduction by IL-18

**Figure 1:**
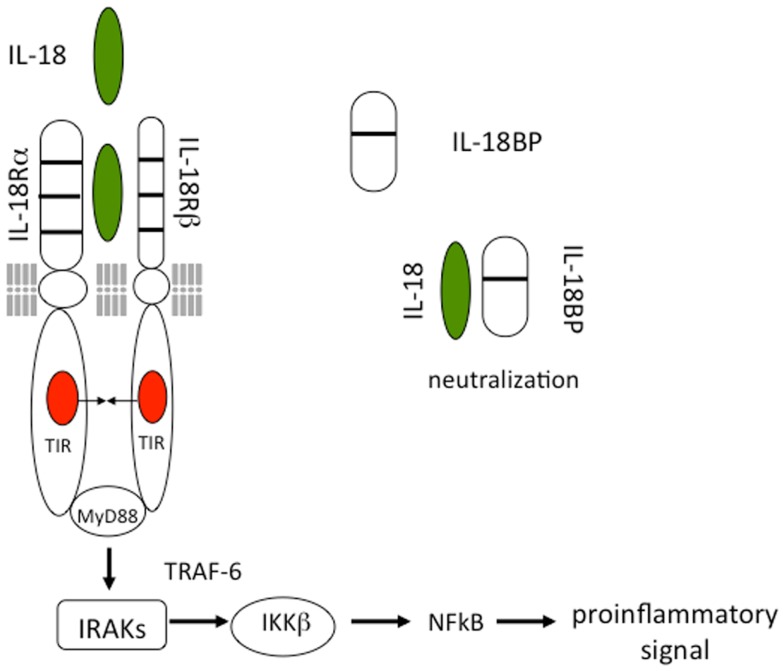
**Interleukin-18 signal transduction**. IL-18 forms a signaling complex by binding to the IL-18 alpha chain (IL-18Rα). The co-receptor, termed IL-18 receptor beta chain (IL-18Rβ), is recruited to form a high affinity complex. Following the formation of the heterodimer, the Toll-IL-1 receptor (TIR) domains approximate triggering the binding of MyD88, phosphorylations of the four IRAKs, TRAF-6, and activation of NFκB. The IL-18BP is present in the extracellular compartment where it binds mature IL-18 and prevents binding to the IL-18 receptor.

As shown in Figure 1, IL-18 forms a signaling complex by binding to the IL-18 alpha chain (IL-18Rα), which is the ligand binding chain for mature IL-18; however, this binding is of low affinity. In cells that express the co-receptor, termed IL-18 receptor beta chain (IL-18Rβ), a high affinity complex is formed, which then signals. The complex of IL-18 with the IL-18Rα and IL-18Rβ chains is similar to that formed by other members of the IL-1 family with the co-receptor, the IL-1R accessory chain IL-1RAcP. Following the formation of the heterodimer, the Toll-IL-1 receptor (TIR) domains approximate and it appears that the cascade of sequential recruitment of MyD88, the four IRAKs and TRAF-6 followed by the degradation of IκB and release of NFκB are nearly identical as that for IL-1 (14). However, there are differences between IL-1 and IL-18 signaling. With few exceptions, IL-1α or IL-1β are active on cells in the low nanograms per milliliter range and often in the picograms per milliliter range. In contrast, IL-18 activation of cells expressing the two IL-18 receptor chains requires 10–20 ng/mL and sometime higher levels (15, 16).

Although nearly all cells express the IL-1RI, not all cells express IL-1RAcP. Similarly, most cells express the IL-18Rα but not all cell express the IL-18Rβ. IL-18Rβ is expressed on T-cells and dendritic cells but not commonly expressed in mesenchymal cells. The human lung epithelial cells line A549, derived from a lung carcinoma epithelial cell, does not express IL-18Rβ (17) and there is no signal unless IL-12 is present to induce IL-18Rβ (18). In the absence of IL-18Rβ, IL-18 binds to IL-18Rα without a pro-inflammatory signal. In A549 cells transfected with IL-18Rβ, IL-18 induces IL-8 and a large number of genes. One of these genes is the former IL-2-induced gene termed NK4 (19) now termed IL-32 (17). IL-32 is not a member of the IL-1 family but plays an important role in the regulation of cytokines such as IL-1β and TNFα. Importantly, IL-32 is an IL-18-inducible gene.

## IL-18 as an Immunoregulatory Cytokine

### Role of IL-18 in the production of IFNγ

Together with IL-12, IL-18 participates in the Th1 paradigm. This property of IL-18 is due to its ability to induce IFNγ either with IL-12 or IL-15. Without IL-12 or IL-15, IL-18 does not induce IFNγ. IL-12 or IL-15 increases the expression of IL-18Rβ, which is essential for IL-18 signal transduction. Importantly, without IL-12 or IL-15, IL-18 plays a role in Th2 diseases (20). The importance of IL-18 as an immunoregulatory cytokine is derived from its prominent biological property of inducing IFNγ from NK cells. Macrophage colony stimulating factor (M-CSF) induces human blood monocytes to differentiate into a subset of macrophages; these cells express a membrane-bound form of IL-18 (21). Membrane IL-18 is expressed in 30–40% of M-CSF-primed macrophages. In contrast, monocytes, dendritic cells, and monocytes differentiated into M1 macrophages did not express membrane IL-18. Although the expression of membrane IL-18 is caspase-1 dependent (21), LPS treatment was necessary for the release of membrane IL-18 (21). A major immunoregulating role for IL-18 is on the NK cell. Upon shedding of membrane IL-18 into a soluble form, NK cells expressed CCR7 and produced high levels of IFNγ. As expected, IFNγ production was prevented by neutralization of IL-18. This mechanism may account for the role of IL-18 as major IFNγ inducing factor from NK cells and the role of NK cells in the pathogenesis of autoimmune diseases.

The induction of IFNγ by IL-18 has been studied with co-inducer IL-12. For example, mice injected with the combination of IL-18 plus IL-12 develop high levels of IFNγ and die with hypoglycemia, intestinal inflammation, and inanition (22). In leptin-deficient mice, IL-18 plus IL-12 induce acute pancreatitis (23). Several human autoimmune diseases are associated with elevated production of IFNγ and IL-18. Diseases such as systemic lupus erythematosus, rheumatoid arthritis, Type-1 diabetes, Crohn’s disease, psoriasis, and graft versus host disease are thought to be mediated, in part, by IL-18.

### IL-18, IL-17, and gamma/delta T-cell activation

The role for IL-17 in the pathogenesis of autoimmune diseases has been studied in animal models but also validated in humans treated with either neutralizing antibodies to IL-17 or the IL-17 receptor. However, blockade of IL-1 often prevents or markedly reduces the production of IL-17 *in vitro* as well as the development of autoimmunity in animal models (24–27). Indeed, there is increased IL-1β as well as increased IL-17 in children born with mutations in the naturally occurring IL-1Ra resulting in a severe inflammatory disease due to excessive IL-1β activity (28, 29). The high production of IL-17 in these children is thought to contribute to the severity of the disease. Is there a role for IL-18 in the production of IL-17?

Attention has focused on a role for IL-18 in Th17 responses primarily because both IL-1β and IL-18 are processed into active cytokines via caspase-1. Using a model for multiple sclerosis termed experimental autoimmune encephalomyelitis (EAE) (26), a role for IL-18 was studied. As expected, using the adjuvant of *Mycobacterium tuberculosis* plus the myelin-derived immunogen for EAE, bone marrow derived mouse dendritic cells released IL-1β and IL-18, which was dependent on caspase-1 (30). The primed dendritic cells induced IL-17 from T-cells, which when transferred to non-immunized mice resulted in the encephalomyelitis. However, the disease did not develop when the dendritic cells were exposed to a caspase-1 inhibitor (30). Treating the mice with either IL-1β or IL-18 restored the ability of the T-cell transfer to induce the disease. Moreover, treating the recipient mice with the caspase-1 inhibitor reduced the disease as well as reduced the production of IL-17 from CD4 positive T-cells as well as from gamma-delta T-cells. Gamma-delta T-cells produce IL-17 when stimulated with IL-18 plus IL-23, as these T-cells express high levels of the IL-18 receptor alpha chain. Thus, similar to caspase-1 dependent IL-1β, IL-18 induces T-cells to produce IL-17 and promote autoimmune responses to specific antigens.

## IL-18 and Inflammation

### Pro-inflammatory properties of IL-18

Interleukin-18 exhibits characteristics of other pro-inflammatory cytokines, such as increases in cell adhesion molecules, nitric oxide synthesis, and chemokine production. Blocking IL-18 activity reduces metastasis in a mouse model of melanoma; this is due to a reduction in IL-18-induced expression of vascular call adhesion molecule-1 (31). A unique property of IL-18 is the induction of FasL, which may account for the hepatic damage that takes place in macrophage activation syndrome (MAS) (10, 32). The induction of fever, a well-studied property of IL-1α and IL-1β as well as acute phase proteins, TNFα and IL-6, is not a significant property of IL-18. Injection of IL-18 into mice or rabbits does not produce fever (33, 34). In a clinical study of intravenous IL-18 dosing in patients with cancer, chills, and fevers were not common and were Grade 1 (low fevers). Unlike IL-1 and TNFα, fever in humans is observed in all patients at doses of 10 ng/kg whereas IL-18 fevers were observed in 3 of 21 patients and only at doses of 100 and 200 μg/kg (35).

Unlike IL-1 and TNFα, IL-18 does not induce cyclooxygenase-2 and hence there is no production of prostaglandin E2 (16, 36). IL-18 has been administered to humans for the treatment of cancer in order to increase the activity and expansion of cytotoxic T-cells. Not unexpectedly and similar to several cytokines, the therapeutic focus on IL-18 has shifted from its use as an immune stimulant to inhibition of its activity (3, 37).

Because IL-18 can increase IFNγ production, blocking IL-18 activity in autoimmune diseases is an attractive therapeutic target since anti-IL-12/23 reduces the severity of Crohn’s disease as well as psoriasis. As discussed below, there appears to be a role for blocking IL-18 in Crohn’s disease. However, there are several activities of IL-18 that are independent of IFNγ. For example, IL-18 inhibits proteoglycan synthesis in chondrocytes (38) and proteoglycan synthesis is essential for maintaining healthy cartilage. IL-18 also increases vascular cell adhesion molecule-1 (VCAM-1) expression in endothelial cells independently of IFNγ. VCAM-1 plays a major role in multiple sclerosis, other autoimmune diseases as well as in the metastatic process (39).

### Role of IL-18 in models of inflammatory bowel disease

Inflammatory bowel disease such as Crohn’s disease is a complex autoimmune disease. Treatment is initially based on immunosuppressive drugs. Not surprisingly, anti-cytokines such as neutralizing monoclonal antibodies to TNFα (40) or to IL-12/23 provide effective treatment for many patients (41, 42). The reduction of IFNγ in Crohn’s disease is linked to the clinical response to these agents (42). IL-18 is found in affected intestinal lesions from Crohn’s disease patients as a mature protein but the IL-18 precursor form is present in uninvolved intestinal tissues (43). This observation was confirmed in a similar assessment of mucosal biopsies from Crohn’s disease patients (44). Antisense RNA to IL-18 decreased IFNγ production in lamina propria mononuclear cells (44).

A commonly used mouse model for colitis is DSS, which is added to the drinking water and which damages the intestinal wall. Thus in DSS-induced colitis, the epithelial barrier defenses against luminal bacterial products are breeched. In this model, reducing IL-18 with a neutralizing antibody is protective and linked to a reduction in IFNγ (9). Blocking IL-18 with the IL-18 binding protein (IL-18BP) (see Figure 1) also reduces colitis induced by antigen sensitization (45). Since generation of active IL-18 requires caspase-1, studies have also been performed in mice deficient in caspase-1 and subjected to DSS colitis. Nevertheless, despite many studies, the role of caspase-1 in DSS colitis remains unclear. The first study showed that mice deficient in caspase-1 were protected (7, 46). In addition, treatment of mice with a specific caspase-1 inhibitor was also effective in protecting against the colitis (47–49). In both studies, the effect of caspase-1 deficiency was linked to reduced IL-18 activity, whereas reducing IL-1 activity with the IL-1Ra was ineffective (7). In support of the role of IL-18 in DSS colitis, inhibition of endogenous merprin β to reduce the generation of active IL-18 was protective in DSS colitis (50).

However, a conundrum has developed whether caspase-1 deficiency is protective or detrimental in DSS colitis. DSS colitis is not the optimal model for Crohn’s disease as the model is one of rapid loss of the protective barrier of the intestinal epithelium exposing the lamina propria mononuclear cells to a large amount and variety of bacterial products. Using the same DSS model, mice deficient in the adapter protein inflammasome component ASC experienced increased disease, morbidity, and precancerous lesions compared to wild-type mice exposed to DSS (51). Similarly, mice deficient in caspase-1 died rapidly from DSS compared to wild-type mice (52) whereas mice deficient in caspase-12, in which caspase-1 is enhanced were protected (52). Administration of exogenous IL-18 restored mucosal healing in caspase-1-deficient mice (52). Also, mice deficient in NLRP3 were more susceptible to either DSS or TNBS-induced colitis and exhibited decreased IL-1β as well as decreased beta-defensins (53). Macrophages from NLRP3-deficient mice failed to respond to MDP (53). Mice deficient in NLRP6 are also more vulnerable to DSS (54, 55) and the susceptibility appears to be due to lack of sufficient IL-18.

How to reconcile these data in mouse models of colitis was addressed by Siegmund (56). It is likely that IL-18, being constitutive in the intestinal epithelium, has a protective role in that the cytokine contributes to maintaining the intestinal barrier. With loss of the barrier, the microbial products stimulate macrophages in the lamina propria and caspase-1 dependent processing of IL-18 results in inflammation. In this model, inhibition of IL-18 production in caspase-1-deficient mice or treatment of wild-type mice with anti-IL-18 antibodies or caspase-1 inhibitors is protective. Worsening of disease in mice deficient in caspase-1 or NLRP3 or NLRP6 may lower the levels of active endogenous IL-18 needed to protect the epithelial barrier. Similarly, active endogenous IL-1β may be needed to protect to maintain the epithelial barrier by inducing growth factors.

Although it remains unclear why caspase-1 deficiency worsens DSS colitis, in humans with Crohn’s disease, natalizumab, the antibody that blocks the very late antigen-4 (VLA-4), is highly effective in treating the disease. VLA-4 is the α4 subunit of the β-1 integrin. Anti-VLA-4 binds to the surface of macrophages and other myeloid cells and prevents the binding of these cells to the VLA-4 receptor on endothelial cells known as VCAM-1. Thus, the antibody disables the function of VCAM-1 and prevents the passage of macrophages and other myeloid cells into tissues such as the intestine in Crohn’s disease and the brain in multiple sclerosis. Since IL-18 induces VCAM-1, blocking IL-18 would also reduce the passage of cells through the endothelium into to intestine.

### IL-18, hyperphagia, and the metabolic syndrome

Whereas there is no constitutive gene expression for IL-1β in freshly obtained human PBMC, the same cells express constitutive mRNA for IL-18 (5). In western blot analysis from the same cells, the IL-18 precursor was present but not the IL-1β precursor. Similar observations were also made in mice (5). These findings suggest that IL-18 may act as regulator of homeostasis. Starting at age 16 weeks of age, IL-18-deficient mice start to overeat, become obese, and exhibit lipid abnormalities; there is increased atherosclerosis, insulin resistance, and diabetes mellitus reminiscent of the metabolic syndrome (57). IL-18Rα deficient mice also develop a similar phenotype. The higher body weight is attributed to enhanced food intake, in which the IL-18-deficient mice begin to diverge from wild-type animals at a relatively early age, and to reach values 30–40% higher than that of wild-type mice. Others have observed similar findings (58). A striking finding was an increase of more than 100% in the percent of adipose tissue in the IL-18-deficient animals that was accompanied by fat deposition in the arterial walls. The insulin resistance in these mice is corrected by exogenous recombinant IL-18. Mice deficient in IL-18 respond normally to a challenge with exogenous leptin suggesting that expression of the leptin receptor is unaffected. The unexpected and unique mechanism is responsible for the higher food intake in the IL-18-deficient animals appears to be due a central nervous system loss of appetite control. IL-18-deficient mice eat throughout the day whereas wild-type mice eat once, nocturnally.

### IL-18 in heart disease

Heart disease includes coronary vessel disease with associated myocardial infarction, post viral myocardiopathies, autoimmune heat disease, and chronic heart failure. Although survival from an acute myocardial infarction has decreased dramatically due to improved acute care, patients often progress to heart failure due to post infarction remodeling of the ventricles. Treatment options for heart failure vary but reducing cytokines is now being tested as a possible therapy. Based on pre-clinical as well as pilot clinical trials, blocking TNFα was tested in large trials but failed; using a higher dose of an antibody to TNFα (infliximab), there were more deaths compared to the placebo-treated patients. There are also pre-clinical studies demonstrating that blockade of IL-1β is effective (59, 60) and clinical trials using anakinra have revealed that blockade of IL-1 is effective in reducing post infarction remodeling (61, 62) as well as increased exercise tolerance (63). In fact, the largest trial in 17,200 patients using a neutralizing antibody to IL-1β aims to reduce cardiovascular events in high risk patients (64).

Increasing numbers of animal and clinical studies indicate a role for IL-18 in heart disease. The myocardium of patients with ischemic heart failure express the alpha chain of the IL-18 receptor and have elevated levels of circulating IL-18 and associated with death (65). Daily administration of IL-18 results in ventricular hypertrophy, increased collagen (66), and elevated left ventricular diastolic pressure in mice (67). As with all cytokine studies, validation of the role of a cytokine in a disease process is best assessed by specific blockade. In a model of myocardial suppression associated with septic shock, mice were injected with LPS and a neutralizing antibody to murine IL-18 was administered (68). The rationale for the experiment was that IL-18 mediates the production of TNFα and IL-1β and to induce the expression of intercellular adhesion molecule-1 (ICAM-1) and VCAM-1. Mice were injected with LPS and left ventricular developed pressure was determined. Left ventricular developed pressure was depressed by 38% 6 h after LPS but pretreatment with anti-mouse IL-18 antibody attenuated LPS-induced myocardial dysfunction (by 92%) and ICAM-1/VCAM-1 expression (50 and 35% reduction, respectively).

In another study, human atrial muscle strips were obtained from patients undergoing by-pass surgery and the tissue was exposed to ischemia while contractile strength was measured. The addition of IL-18BP to the perfusate during and after the ischemic event resulted in improved contractile function from 35% of control to 76% with IL-18BP (69). IL-18BP treatment also preserved intracellular tissue creatine kinase levels (by 420%). Steady-state mRNA levels for IL-18 were elevated after ischemic and the concentration of IL-18 in myocardial homogenates was increased (control, 5.8 pg/mg versus I/R, 26 pg/mg). Active IL-18 requires cleavage of its precursor form by caspase-1; inhibition of caspase-1 also attenuated the depression in contractile force after ischemia (from 35% of control to 75.8% in treated atrial muscle). Because caspase-1 also cleaves the IL-1β precursor, IL-1 receptor blockade was accomplished by using the IL-1 receptor antagonist. IL-1 receptor antagonist added to the perfusate also resulted in a reduction of ischemia-induced contractile dysfunction.

In summary, these studies demonstrate a role for IL-18 in heart disease. Moreover, endogenous IL-18 is induced by IL-1β via caspase-1 under ischemic conditions in human myocardial tissue and that inhibition of caspase-1 reduces the processing of endogenous precursors of IL-18 and IL-1β and thereby prevents ischemia-induced myocardial dysfunction.

## IL-18 as a Protective Cytokine

As stated above, mice deficient in caspase-1 experience increased disease severity when subjected to DSS colitis and that administration of exogenous IL-18 restored mucosal healing in these mice (52). In addition, IL-18 deficiency or IL-18 receptor deficiency results in the development of a metabolic syndrome in mice. Mice deficient in NLRP3 are more susceptible to DSS colitis, which is thought to be due to decreased IL-18 (53). Mice deficient in NLRP6 are also more vulnerable to DSS (54, 55) and the susceptibility appears to be due to lack of sufficient IL-18. As discussed below, a protective role for IL-18 is not limited to the gastrointestinal track. In the eye, a condition resembling “wet macula degeneration” worsens with antibodies to IL-18 (70).

Thus, there are a growing number of studies, which support a protective role for IL-18. The fact that mice deficient in IL-18 develop a metabolic syndrome-like phenotype is consistent with a role for IL-18 in homeostasis. A study in age related macular degeneration is also consistent with a protective role for IL-18. In that study, drusen, which is mixture of complement-derived and apolipoproteins and lipids, were shown to activate NLRP3 and induce the production of mature IL-1β and IL-18 (70). In a mouse model of “wet” age related macular degeneration, the disease was worse in mice deficient in NLRP3 but not in IL-1RI deficient mice (70). Therefore, IL-18 rather than IL-1α or IL-1β were protective and upon administration of IL-IL-18, the disease severity improved. Taken together, there is a case for IL-18 being a protective rather than inflammatory cytokine.

## IL-18 Binding Protein

### The discovery of the IL-18BP

The discovery of the IL-18BP took place during the search for the soluble receptors for IL-18 (71). IL-18BP is a constitutively secreted protein, with an exceptionally high affinity for IL-18 (400 pM) (72) (Figure 1). Present in the serum of healthy humans at a 20-fold molar excess compared to IL-18 (73), IL-18BP may contribute to a default mechanism by which a Th1 response to foreign organisms is blunted in order to reduce triggering an autoimmune responses to a routine infection. IL-18BP deviates from the classical definition of soluble receptors since it does not correspond to the extracellular ligand binding domain of the IL-18 receptor, but is rather encoded by a separate gene. Thus IL-18BP belongs to a separate family of secreted proteins. As shown in Figure 1, IL-18BP contains only one IgG domain whereas the Type II IL-1 receptor contains three domains. In this regard, the single IgG domain of IL-18BP is similar to SIGIRR, which also has a single IgG domain and also functions as a decoy receptor. The salient property of IL-18BP in immune responses is in down-regulating Th1 responses by binding to IL-18 and thus reducing the induction of IFNγ (20). Since IL-18 also affects Th2 responses, IL-18BP also has properties controlling a Th2 cytokine response (20).

### Balance of IL-18 and IL-18BP in human disease

IL-18 binding protein has a classic signal peptide, and therefore is readily secreted. Serum levels in healthy subjects are in the range of 2,000–3,000 pg/mL compared to the levels of IL-18 in the same sera of 80–120 pg/mL (73). Moreover, IL-18BP binds IL-18 with an affinity of 400 pM, an affinity significantly higher than that of IL-18Rα. Because a single IL-18BP molecule binds a single IL-18 molecule, one can calculate bound versus free IL-18 in a mixture of both molecules (73).

If one examines immunologically mediated diseases where IFNγ plays a pathological role such as Wegener’s granulomatosis and systemic lupus erythematosus, one must consider the level of free IL-18 compared to IL-18 bound to IL-18BP. In fact, in these diseases both IL-18BP and IL-18 are high (74, 75) but the level of IL-18BP is not sufficiently high enough to neutralize IL-18 and therefore, the level of free IL-18 is higher than in healthy subjects. In MAS where IFNγ plays a pathological role, both IL-18BP and IL-18 are also high but the clinical and hematological abnormalities correlate with elevated free IL-18 (32).

A unique property of IL-18BP is that the molecule also binds IL-37 (76) and in doing so, enhances the ability of IL-18BP to inhibit the induction of IFNγ by IL-18. IL-37 binds to the IL-18Rα with a very low affinity but in mice expressing human IL-37, a profound anti-inflammatory effect is observed (77), particularly of LPS-induced cytokines and dendritic cell maturation (77). Human IL-37-expressing mice are also resistant to colitis (78). Thus, the anti-inflammatory property of IL-37 can be affected by the concentration of IL-18BP. As the concentration of IL-18BP increases and binds IL-37, there is the possibility that IL-37 becomes less available as an anti-inflammatory cytokine. Indeed this has been observed in mice injected with IL-18BP. At low dosing of IL-18BP, there is reduced inflammation in a model of rheumatoid arthritis but as the doing of IL-18BP increases, the anti-inflammatory properties of IL-18BP are lost (79). Table 1 summarizes several disease states in which IL-18 as well as IL-18BP are measured and in some studies, the level of free IL-18 has been reported.

**Table 1:** **Levels of IL-18 and IL-18BP in human disease**.

Disease	IL-18^a^	IL-18BP^b^	Free IL-18^a^	Reference
Sepsis	500–2,000	ND	ND	Emmanuilidis et al. (100 )
Sepsis	250–10,000	22.5	250–3,000	Novick et al. (73 )
Trauma	300–600	ND	ND	Mommsen et al. (101 )
Schizophrenia	518	10	253	Palladino et al. (102 )
Ulcerative colitis	274	ND	ND	Haas et al. (103 )
Ulcerative colitis	393	4.7	250	Ludwiczek et al. (104 )
Crohn’s disease	387	ND	ND	Haas et al. (103 )
Crohn’s disease	546	5	340	Ludwiczek et al. (104 )
Wegener’s disease	240	14.5	84	Novick et al. (74 )
Rheumatoid arthritis	230–400	ND	ND	Bokarewa and Hultgren (105 )
SLE^c^	700	7.5	408	Favilli et al. (99 )
SLE^c^	400	15	167	Novick et al. (75 )
MAS^d^	2,200	35	660	Mazodier et al. (32 )
Systemic JIA^e^	1,600–78,000	ND	ND	Jelusic et al. (106 )
Adult Still’s disease	1,000–6,000	ND	ND	Kawashima et al. (107 )
Myocardial infarction	238	ND	ND	Blankenberg et al. (108 )
Myocardial infarction	355	ND	ND	Narins et al. (109 )
Coronary artery disease	356	13.7	125	Thompson et al. (110 )
Metabolic syndrome	380	ND	ND	Troseid et al. (111 )
Acute kidney injury^f^	500	ND	ND	Parikh et al. (112 )
Acute kidney injury^f^	2,000	ND	ND	Vaidya et al. (113 )
Acute kidney injury^f^	>360	ND	ND	Parikh et al. (114 )
Acute kidney injury^f^	884	ND	ND	Sirota et al. (115 )

^a^Levels in picograms per milliliter, range, or mean.

^b^Levels in nanograms per milliliter, range, or mean.

^c^Systemic lupus erythematosus.

^d^Macrophage activation syndrome.

^e^Systemic juvenile idiopathic arthritis.

^f^Urine levels (mean in picograms per milliliter).

### Regulation of IL-18BP

IL-18 binding protein is highly regulated at the level of gene expression and unexpectedly, IFNγ increases gene expression and synthesis of IL-18BP (80, 81). Therefore, IFNγ driving an increase in the natural and potent inhibitor of IL-18 falls into the category of a negative feed-back loop. The concept is supported by clinical data showing that patients being treated with IFNα for hepatitis have elevated levels of IL-18BP (82, 83). IL-27, like IFNγ, functions as both a pro- as well as an anti-inflammatory cytokine and both may accomplish their roles as anti-inflammatory cytokines at the level of increased production of IL-18BP. In the skin, IL-27 also acts through a negative feed-back loop for inflammation. IL-27 is acting, as is IFNγ, by induction of IL-18BP gene expression and synthesis (84).

### Viral IL-18BP

Natural neutralization of human IL-18 by IL-18BP takes place during a common viral infection. In *Molluscum contagiosum* infection, characterized by raised but bland eruptions, there are large numbers of viral particles in the epithelial cells of the skin but histologically there are few inflammatory or immunologically active cells in or near the lesions. Clearly, the virus fails to elicit an inflammatory or immunological response. Amino acid similarity exists between human IL-18BP and a gene found in various members of the poxviruses; the greatest degree of homology is found to be expressed by *M. contagiosum* gene (85). The ability of viral IL-18BP to reduce the activity of mammalian IL-18 likely explains the lack of inflammatory and immune cells in the virally infected tissues and provides further evidence for the natural ability of IL-18BP to interfere with IL-18 activity.

## Hemophagocytic Lympho Histiocytosis and Macrophage Activation Syndrome

Hemophagocytic lympho histiocytosis (HLH) syndrome is a rare life-threatening condition characterized by a severe hyper-inflammatory state. There is a genetic form of HLH called familial hemophagocytic lympho histiocytosis (fHLH). However, HLH can be secondary to infections and lymphoma, and is called secondary MAS. The development of MAS is associated with several infectious diseases, notably due to Epstein–Barr virus, cytomegalovirus, herpes virus, or intracellular bacteria and parasites and also of various lymphomas, especially of T-cell lineage. In addition, patients with rheumatological conditions, particularly systemic onset juvenile arthritis (sJIA), but also systemic lupus erythematosus, Kawasaki disease, or systemic vasculitis can develop MAS (86–89). One of the most prominent hematologic and metabolic characteristics of MAS is thrombocytopenia and hepatic injury, respectively. Indeed, IFNγ may be responsible for the thrombocytopenia as well as several of the immunological abnormalities of the disorder.

## IL-18 in the Hemophagocytic Syndromes

In the case of fHLH or MAS, gene expression for IL-18 is up-regulated in peripheral mononuclear cells (90, 91) and serum IL-18 is unusually elevated (32, 92– 95). Although levels of IL-18 in the circulation are below 1 ng/mL in inflammatory diseases such as severe sepsis, in active phase of fHLH or EBV-HLH, serum IL-18 is usually in the range of 5–7 ng/mL, and in fHLH complicating XIAP gene mutations as well as in MAS complicating sJIA, levels of circulating IL-18 can be in 20–30 ng/mL range (32, 96– 98). However, it is necessary to calculate the level of free IL-18 since IL-18BP is present in the circulation in health and disease (73) (see Table 1) in lupus (75, 99), Wegener’s granulomatosis (74). In patients with MAS, free IL-18 but not IL-12 concentrations significantly correlated with clinical status and the biologic markers of MAS such as anemia (*p* < 0.001), hypertriglyceridemia, and hyperferritinemia (*p* < 0.01) and also with markers of Th1 lymphocyte or macrophage activation, such as elevated concentrations of IFNγ and soluble IL-2 and TNFα receptor concentrations (32).

## Concluding Remarks

Although clinical trials of IL-1 blocking therapies have focused attention on the biology IL-1, the role of IL-18 in health and disease is derived from animal models and measurements of IL-18 in various disease conditions. Nevertheless, with clinical trials of IL-18BP as well as neutralizing antibodies to IL-18 now underway, the role for this cytokine in treating human disease will become apparent. Certainly validated animal models support a role for IL-18 in acute renal injury, psoriasis, heart failure, MAS, and inflammatory bowel disease. Whether suppression of IL-18 will affect IL-17-mediated diseases such as multiple sclerosis or reduce metastatic melanoma will also be determined in clinical trials.

## Conflict of Interest Statement

The authors declare that the research was conducted in the absence of any commercial or financial relationships that could be construed as a potential conflict of interest.
